# Platelet glycoprotein Ibα is an important mediator of ischemic stroke in mice

**DOI:** 10.1186/2040-7378-3-9

**Published:** 2011-09-13

**Authors:** Simon F De Meyer, Tobias Schwarz, Daphne Schatzberg, Denisa D Wagner

**Affiliations:** 1 Immune Disease Institute, (3 Blackfan Circle), Boston, (MA 02115), USA; 2 Program in Cellular and Molecular Medicine, Children's Hospital Boston, (300 Longwood Avenue), Boston, (MA 02115), USA; 3 Department of Pediatrics, Harvard Medical School, (25 Shattuck Street), Boston, (MA 02115), USA; 4 Laboratory for Thrombosis Research, KULeuven Campus Kortrijk, (E. Sabbelaan 53), Kortrijk, (B-8500), Belgium; 5 Department of Neurology, University of Wuerzburg, (Josef-Schneider-Str. 11), Wuerzburg, (D-97080), Germany

## Abstract

**Background:**

Platelets play an important role in ischemic stroke. GPIbα is a major platelet receptor that is critical for platelet adhesion to exposed subendothelial matrix components at sites of vascular damage.

**Methods:**

In this study, we used transgenic mice in which the extracellular part of GPIbα is replaced by human interleukin 4-receptor (GPIbα/IL4Rα). We observed normal brain vasculature in these mice. We compared infarct size in GPIbα/IL4Rα and wild-type (WT) mice 23 hours after 1-hour transient middle cerebral artery occlusion (tMCAO). In addition, the functional outcome was evaluated using a modified Bederson score.

**Results:**

We found a significantly smaller infarct size in GPIbα/IL4Rα mice compared to WT mice (38.0 ± 6.5 mm^3 ^vs. 74.2 ± 8.6 mm^3^, p < 0.001). The decrease in infarct size was functionally relevant as indicated by a significantly better functional Bederson score in GPIbα/IL4Rα mice compared to WT animals (1.3 ± 0.4 vs. 2.7 ± 0.3, p < 0.05).

**Conclusions:**

Our data illustrate and further confirm the important role of platelet GPIbα in ischemic stroke, suggesting that targeted inhibition of this receptor may open new avenues in stroke treatment.

## Background

Stroke is one of the leading causes of death worldwide with limited treatment options [1]. Platelets play a pivotal role in cerebral ischemia/reperfusion injury by adhering to the damaged vessel wall, leading to further platelet recruitment and thrombus formation. The glycoprotein (GP) Ib-IX-V complex is a crucial platelet receptor for initial tethering and adhesion at sites of vascular injury. This abundant complex on the platelet surface (12,500 copies per cell) consists of the leucine-rich repeat glycoproteins GPIbα, GPIbβ, GPIX and GPV in a 2:2:2:1 ratio [2]. The adhesive function of GPIb-IX-V is mainly attributed to the interaction of GPIbα with its major ligand von Willebrand factor (VWF), exposed upon vascular damage. The central role of the GPIbα-VWF interaction in mediating initial platelet adhesion is illustrated by the bleeding disorders Bernard Soulier syndrome [3] and von Willebrand disease [4], caused by deficiency of GPIb-IX-V or VWF respectively. Besides its interaction with VWF, GPIbα can also engage counter-receptors such as αMβ2 (Mac-1) on neutrophils and P-selectin on activated platelets or endothelial cells [2]. Other GPIbα ligands include α-thrombin, clotting factors XI and XII, thrombospondin-I and high molecular weight kininogen [2]. Not surprisingly, the importance of GPIbα far exceeds that of VWF in arterial thrombosis [5]. Thus, by binding a variety of ligands, GPIbα is a central receptor in different vascular processes of thrombosis and inflammation, all of which may contribute to the progression of ischemic stroke. Here, we studied stroke development in transgenic mice expressing GPIbα in which the extracellular domain was replaced by an isolated domain of the α-subunit of the human IL-4 receptor [6]. We found that these mice had better stroke outcome, as evidenced by smaller infarct volumes and better functional scores.

## Methods

### Animals

GPIbα/IL4Rα mice [6] and wild-type (WT) type mice (Jackson Laboratory, Bar Harbor, ME) were 8-10 weeks old males on C57BL/6J background. All experimental procedures were approved by the Animal Care and Use Committee of the Immune Disease Institute (Boston, USA).

### Assessment of the cerebral vasculature

For assessment of the cerebral vasculature, animals were deeply anesthetized with isoflurane and transcardially perfused with phosphate buffered saline, followed by 5 ml of black ink. Brains were carefully removed, fixed in 4% PFA and the Circle of Willis and major arteries were examined under a dissecting microscope. The development of the posterior communicating arteries (PComAs) was examined and scored as described [7].

### Platelet counts

To measure platelet counts, blood was collected on EDTA using coated capillaries via retro-orbital puncture. Platelet count in whole blood was determined using a Beckman Coulter AcT Diff 2 Hematology analyzer.

### Induction of cerebral ischemia

Focal cerebral ischemia was induced by 60 min transient middle cerebral artery occlusion (tMCAO) as described [8-10]. Mice were anesthetized with 2% isoflurane/oxygen mixture. Following a midline skin incision in the neck, the proximal common carotid artery, and the external carotid artery were ligated, and a standardized silicon rubber-coated 6.0 nylon monofilament (6021; Doccol Corp., Redlands, CA) was inserted and advanced via the right internal carotid artery to occlude the origin of the right MCA. Operation time per animal did not exceed 15 minutes. The intraluminal suture was left *in situ *for 60 minutes. Then, animals were re-anesthetized, and the occluding monofilament was withdrawn to allow reperfusion.

Some animals were exclusively used for laser-Doppler flowmetry (Periflux 5000, Perimed, Kings Park, NY) to monitor regional cerebral blood flow (rCBF) in the MCA territory (6 mm lateral and 2 mm posterior from bregma).

Mice were excluded from analysis when death occurred within 24 h after tMCAO or when subarachnoid hemorrhage was mascroscopically observed during brain harvesting. No difference in exclusion rates between the two groups was observed.

### Assessment of infarct volume

Mice were sacrificed 24 hours after tMCAO. Brains were quickly removed and cut into 2-mm-thick coronal sections using a mouse brain slice matrix. The slices were stained with 2% 2,3,5-triphenyl-tetrazolium chloride (TTC; Sigma-Aldrich, St. Louis, MO) in PBS to visualize the infarctions. Sections were photographed with a digital Nikon D70 camera and infarct areas (white) were measured by a blinded rater using Image J software (National Institutes of Health; http://rsbweb.nih.gov/ij).

### Assessment of functional outcome

Neurological function was assessed, blinded for the mouse genotype, 24 h after tMCAO, using the modified Bederson score [11]. This test determines global neurological function according to the following scoring system: 0, no deficit; 1, forelimb flexion; 2, decreased resistance to lateral push; 3, unidirectional circling; 4, longitudinal spinning; 5, no movement.

### Statistical analysis

For statistical analysis the unpaired 2-tailed t-test was used (PrismGraph 4.0 software package, La Jolla, CA). The Mann-Whitney U-test was used for analyzing the Bederson score. P values less than 0.05 were considered statistically significant. Results are shown as mean ± SD.

## Results

### GPIbα/IL4Rα and WT animals have comparable cerebral vasculature and regional cerebral blood flow

**Figure 1:**
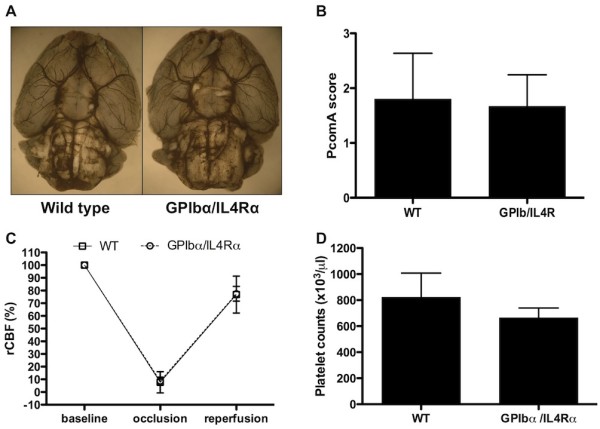
**Regional cerebral blood flow and cerebral vasculature in WT and GPIbα/IL4Rα mice**. (A) Representative stainings of cerebral vasculature by black ink perfusion of WT and GPIbα/IL4Rα mice. (B) Score assessing the posterior communicating arteries perfusion of WT (n = 5) and GPIbα/IL4Rα mice (n = 3). (C) Regional cerebral blood flow of the right middle cerebral artery territory of WT (n = 5) and GPIbα/IL4Rα mice (n = 3), measured at baseline, during occlusion and 15 min after reperfusion (reperfusion). (D) Platelet counts of WT (n = 9) and GPIbα/IL4Rα mice (n = 6). No statistically significant differences were observed in all parameters (A-D).

Upon assessment of the cerebral vasculature in WT (n = 5) and GPIbα/IL4Rα (n = 3) mice, no major anatomic differences were observed which could influence stroke outcome (Figure 1A). The circle of Willis and the distribution of the MCA trunk and branch appeared to be anatomically identical between the genotypes. In addition, the score assessing formation of the posterior communicating arteries of both hemispheres, which can influence susceptibility to tMCAO, did not differ significantly (1.8 ± 0.4 for WT versus 1.7 ± 0.3 for GPIbα/IL4Rα mice, p = 0.82, Figure 1B).

To compare rCBF in the right MCA territory, laser doppler flowmetry in WT (n = 5) and GPIbα/IL4Rα (n = 3) was monitored at baseline levels, after insertion of the occluding filament (ischemia) and 15 minutes after removal of the filament (reperfusion). After advancing the filament, the decrease in rCBF was similar between WT and GPIbα/IL4Rα animals, indicating comparable occlusion of the MCA origin (7.8 ± 4.8% of baseline level in WT mice versus 8.8 ± 1.8% of baseline level in GPIbα/IL4Rα mice, p = 0.88, Figure 1C). Fifteen minutes after reperfusion, rCBF was reconstituted in all animals to > 70% of baseline levels and again did not significantly differ between WT and GPIbα/IL4R animals (80.9 ± 6.6% of baseline level in WT mice versus 77.4 ± 3.4% of baseline level in GPIbα/IL4Rα mice; p = 0.69, Figure 1C). Platelet counts of GPIbα/IL4Rα mice (664 ± 31 × 10^3^/μl, n = 6) were slightly lower than in WT animals (822 ± 62 × 10^3^/μl, n = 9) although this difference was not significant (p = 0.071, Figure 1D).

### GPIbα/IL4Rα animals have a better outcome after tMCAO

**Figure 2:**
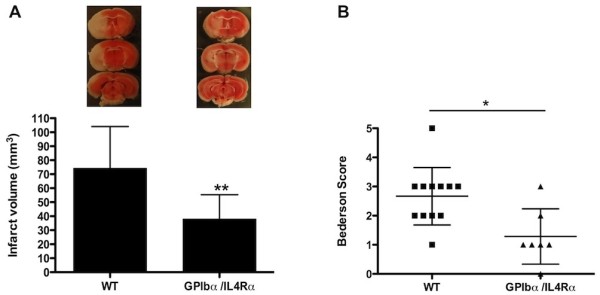
**Infarct volumes and functional outcomes 23 hours after transient middle cerebral artery occlusion in WT and GPIbα/IL4Rα mice**. (A) Representative 2,3,5-TTC stains of 3 corresponding coronal brain sections of wild type (left) and GPIbα/IL4Rα (right) mice 23 h after tMCAO (top) and brain infarct volumes of the 2 groups as measured by planimetry 23 h after tMCAO (bottom, n = 12 and 7 respectively). (B) Neurologic Bederson score of WT and GPIbα/IL4Rα mice as assessed at day 1 after tMCAO (n = 12 and 7 respectively) ** *p <*.001, *p <.05.

To investigate the role of GPIbα-mediated interactions in ischemic stroke, infarct sizes of GPIbα/IL4Rα (n = 7) and WT mice (n = 12) were compared after one hour tMCAO and 23 hours of reperfusion. Interestingly, we found that GPIbα/IL4Rα mice had a ~50% reduction in infarct size compared to WT animals (38.0 **± **6.5 mm^3 ^versus 74.2 **± **8.6 mm^3^, p < 0.05, Figure 2A). The decrease in infarct size was functionally relevant as shown by a significantly better outcome in the Bederson test for the GPIbα/IL4Rα mice compared to WT mice (1.3 **± **0.4 versus 2.7 **± **0.3, p < 0.05, Figure 2B). Taken together, these results indicate that the GPIbα receptor is critically involved in cerebral ischemia/reperfusion injury during stroke.

## Discussion

We here report that mice lacking the extracellular part of GPIbα are protected from ischemic stroke as evidenced by 50% smaller infarct volumes and significantly better neurologic outcome when compared to WT mice. These results further establish the notion that GPIbα is critically involved in the pathogenesis of ischemic stroke [12,13]. Our findings are in agreement with an earlier study using the same mouse tMCAO model in which an anti-GPIbα monoclonal antibody was used to block the GPIbα receptor [14]. Use of this antibody led to a 60% reduction of infarct volumes compared to control mice, which was accompanied by a significant reduction in neurologic deficits [14]. We recently demonstrated that VWF deficient mice were protected from ischemic stroke [8,15]. Since VWF is the main ligand of GPIbα on platelets, our data support the notion of a pathophysiological role for the GPIbα-VWF interaction in stroke, although we can not rule out that other GPIbα-mediated interactions are also important and abrogated in GPIbα/IL4Rα mice. In full support of this, we showed that VWF-deficient mice that were reconstituted with a VWF mutant defective in binding to GPIbα retained their protection against stroke [9]. Moreover, several conditions, including the GPIbα Kozak polymorphism [16], GPIbα variants with increased binding to VWF [17] and high VWF levels [18,19], have all been reported to be associated with an increased risk of ischemic stroke in humans.

It has to be noted that GPIbα/IL4Rα mice were reported to have slightly reduced platelet counts [6]. Although not significant, also our measurements indicate a slightly lower platelet count in GPIbα/IL4Rα mice compared to WT animals. Whereas we cannot completely exclude any effect of this small platelet count difference on stroke outcome, we believe that this is unlikely to cause the observed dramatic protection against stroke. Platelet adhesion to ferric chloride-treated mesenteric arterioles was virtually absent in GPIbα/IL4Rα mice, resulting in the complete abrogation of thrombus formation [5]. VWF-deficient mice are still able to form thrombi in this model, although thrombus formation is significantly delayed [20,21]. Although these studies showed that ligands other than VWF can support GPIbα-dependent platelet adhesion, interfering with the binding of GPIbα to VWF has indeed been proven to have a profound antithrombotic effect [22-24]. Since an increasing amount of evidence suggests that GPIbα and VWF could also mediate inflammatory processes, further studies are needed to establish whether inhibition of these molecules also reduce inflammation in stroke [25].

The search for safe and efficient drugs for stroke management has been hampered by an unacceptable risk of fatal bleeding as seen e.g. with platelet aggregation inhibitors [26]. In this respect, it is encouraging that we did not observe intracranial hemorrhages after stroke in GPIbα/IL4Rα mice. Also in VWF deficient mice, no increased bleeding was noticed in the tMCAO model [8,9,15]. In comparison with inhibition of platelet aggregation, neutralizing the VWF-GPIbα-axis has been shown to have a better benefit-to-risk ratio with respect to bleeding in various models of experimental thrombosis [22-24]. Interestingly, a recent trial showed that administration of anti-VWF aptamer ARC1779, which blocks the VWF-GPIbα interaction, reduced cerebral emboli signals in patients undergoing carotid endarterectomy [27].

## Conclusions

In conclusion, using GPIbα/IL4Rα mice, we demonstrated the crucial role for GPIbα in ischemic stroke. Together with the already existing evidence, our results further warrant larger translational and clinical studies to assess the benefits or drawbacks of interfering with platelet adhesion in stroke.

## Competing interests

The authors declare that they have no competing interests.

## Authors' contributions

SFDM conceived the study, conducted experiments and wrote the paper. DS conducted experiments and took care of mouse breeding. TS provided the tMCAO expertise. DW conceived and funded the study and revised the paper. All authors read and approved the final manuscript.
